# Environmental Concern Priming and Social Acceptance of Sustainable Technologies: The Case of Decentralized Wastewater Treatment Systems

**DOI:** 10.3389/fpsyg.2021.647406

**Published:** 2021-09-16

**Authors:** Cristina Gómez-Román, José-Manuel Sabucedo, Mónica Alzate, Beatriz Medina

**Affiliations:** ^1^Department of Social Psychology, Basic and Methodology, CRETUS Institute, Universidade de Santiago de Compostela, Santiago de Compostela, Spain; ^2^Department of Social Psychology, Basic and Methodology, Universidade de Santiago de Compostela, Santiago de Compostela, Spain; ^3^Water, Environment and Business for Development (WE&B), Barcelona, Spain

**Keywords:** environmental concern priming, type of information, social acceptance, sustainable technologies, decentralized wastewater treatment plants, water problems

## Abstract

According to a report by the World Economic Forum, the water crisis is the fourth most serious global risk to society. The apparent limitations of the hydraulic paradigm to solving this crisis are leading to a change in water management approaches. Recently, decentralized wastewater treatment systems have re-emerged as a partial solution to this problem. However, to implement these systems successfully, it is necessary not only to design this technology but also to have social support and willingness among citizens to use it. Previous studies have shown that these technologies are often perceived as being too costly, and people often do not consider the need for adopting them. However, it has also been pointed out that thinking about these technologies as a sustainable endeavor to reduce human impact on the environment can help to overcome the barriers to usage. Thus, we test whether priming environmental concerns before presenting information about decentralized wastewater treatment plants will increase acceptance of those technologies. In this study, we test whether priming environmental concerns can enhance the acceptance of decentralized wastewater treatment plants even when presenting disadvantages of the technology. In order to do so, we designed an experimental study with a sample of 287 people (85.7% women, *M*_age_=20, 28). The experimental design was 2 (priming the environmental concern vs. no priming)×2 (type of information: only advantages vs. advantages and disadvantages). The results showed that those in the environmental concern priming condition had more positive attitudes and behavioral intentions toward decentralized wastewater treatment plants than those in the control condition group. Participants who received only advantages information had a more positive perception toward the decentralized wastewater systems than in the condition, where disadvantages were present, but in the priming condition this difference was not significant. This implies that priming environmental concern helps to overcome the possible disadvantages that act as barriers to acceptance.

## Introduction

According to the World Economic Forum (2020), the water crisis is one of the top five global problems. The water crisis is related to both the scarcity of this resource and its quality due to pollution and eutrophication (Ganoulis, 2009; World Water Assessment Programme, 2020). Solving this crisis depends partly on changing people’s behavior. Various campaigns have tried to reduce water consumption and make the population aware of the limited nature of this resource (Syme et al., 2000; Katz et al., 2016). However, the demand for water continues to increase. For this reason, the United Nations warns that there is an urgent need to address the crucial challenges caused by water stress, since current water management is failing to respond to this problem (Cosgrove and Loucks, 2015; Seemha and Ganesapillai, 2017).

An alternative approach to address this crisis is to use technologies that facilitate the reuse of water (Fielding et al., 2018) and better use of the nutrients in wastewater, thus preventing untreated waste from causing the deterioration of freshwater resources (Lam et al., 2020). One such technology is decentralized wastewater treatment plants. This technology challenges the current approach to disposing of waste far from home; it involves local treatment of wastewater (in buildings, neighborhoods, or small communities), favoring the local recovery of water and nutrients for new uses, thus promoting the circular economy (Lens et al., 2005; Roefs et al., 2017).

Nevertheless, despite the advantages of decentralized plants, they can also result in installation, maintenance, and location-based costs (Mankad and Tapsuwan, 2011). Therefore, people may be reluctant to install this type of technology unless advantages over the current centralized system are apparent. In other words, the traditional resistance to change (Petty et al., 2003) could be present in this case. Some may have a reactive response to a technology that is unfamiliar, externally imposed, and may have unclear implications from their perspectives. This is especially prevalent in places where water issues, and environmental sustainability more generally, are not perceived as a problem (Gómez-Román et al., 2020).

Given this situation, providing information to citizens can improve acceptance of these technologies (Mankad and Tapsuwan, 2011; Mankad, 2012). However, what kind of information will have the most impact on social acceptance? To answer this question, there are two important things one must consider. On the one hand, what is the level of concern about the issue that this technology aims to solve? On the other hand, what kind of information should be offered to the public about the new technology?

### Environmental Concern

Decentralized plants serve as an alternative to an environmental problem: water stress. Therefore, a necessary – although not sufficient – condition for the acceptance of that technology is the existence of some public awareness or concern about environmental issues. If the public does not feel environmental issues are a problem, strategies to solve the problem of water stress will receive little or no support.

In recent years, concern about environmental issues has grown significantly (Liu et al., 2014; Currie and Choma, 2018; Lewis et al., 2019). Environmental policymaking has become part of the agenda of nearly all political bodies around the world (Krosnick et al., 2006; Fairbrother, 2017), and it is also a subject on which there is broad social consensus (Steg and Vlek, 2009; Eurobarometer, 2019). All of these favor the acceptance of environmental sustainability and circular economy policies. Studies on the perception of environmental risk clearly show how concern for the environment is one of the antecedents of pro-environmental attitudes and behaviors (O’Connor et al., 1999, 2002; Heath and Gifford, 2006; Hidalgo and Pisano, 2010).

In accordance with the above, the activation and accessibility of the environmental issue, insofar as it evokes the problems in this area, could translate into attitudes, emotions and behaviors more favorable to decentralized plants; that is, in a greater acceptance of this technology. This leads us the concept of priming. Studies on priming analyze how exposure to prior information affects a subsequent decision or behavior (Jonas and Sassenberg, 2006; Custers and Aarts, 2010). So, according to this, making accessible or priming the environmental concern could make more accessible information that already exists in memory or associated processes (the environmental problem), so that it becomes salient in subsequent decision-making (Kay et al., 2004; Scheufele and Tewksbury, 2007), in this case being more favorable to accept decentralized plants.

Nevertheless, in addition to concern for the environment, which in this case would be activated through priming, there are other possible factors involved in the acceptance of decentralized wastewater treatment plants. Among them, the cost–benefit calculation is of importance; here, it includes not only economic issues but also elements such as loss of comfort or aspects related to technology maintenance (Mankad and Tapsuwan, 2011).

### Information About Technology: Focusing Only on the Positive?

As discussed previously, providing the population with information assists in overcoming barriers to acceptance (Mankad and Tapsuwan, 2011; Fielding et al., 2018), especially in places where public opinion has not yet been able to form an impression about it (Jacoby, 2000). However, when presenting information to the public, one must take into account that several elements may influence (to a greater or lesser extent) the effect that information may have. One of these factors is related to the unilateral or bilateral nature of the arguments presented to the public. The first consists of expressing only the advantages and positive aspects, while the latter also includes weak or negative aspects of a technology.

There is mixed evidence on the efficacy of presenting unilateral or bilateral arguments (Allen, 1991). The effectiveness of including disadvantages in persuasive messaging is not entirely clear, especially when the public does not yet have an elaborate opinion on the subject under study (Rosenberg, 2001). Presenting positive messages while also discussing some disadvantages or less positive elements improves source credibility, and the public may have more confidence in the veracity of such messaging (Crowley and Hoyer, 1994; Schlosser, 2011). Nevertheless, the persuasiveness of messaging will also depend on whether the disadvantages that are presented (and refuted) are relevant to the people receiving the message (O’Keefe, 1990). The effect may also be different depending on the recipient of the message. One-sided messages (unilateral) appear to be more effective when the audience is initially in favor of the message’s content (Petty and Cacioppo, 1986). However, if the recipients are well-informed, two-way (bilateral) messages are more effective.

### Study Aims and Hypothesis

This exploratory study analyzes the influence that environmental concern priming and different types of messages (unilateral vs. bilateral) about decentralized plants have on the social acceptance of this sustainable technology. Our hypotheses are as follows:

H_1_: The activation of environmental concern priming will favor the acceptance of decentralized wastewater plants.

H_2_: Public perception of decentralized plants will be more positive when the information presented relates only to the plant’s advantages.

H_3_: An interaction between priming and information will occur when messaging relates only to the plant’s advantages or advantages and disadvantages. When environmental concern priming is not activated, public perception of the plants will be most negative when discussing the disadvantages (as opposed to the condition where only advantages are discussed). However, there will be no significant difference in technology acceptance between participants when the environmental concern priming is activated (irrespective of presentation of the advantages or both advantages and disadvantages).

## Materials and Methods

### Participants and Design

Using GPower software (v 3.1.9.4), a power analysis was conducted to calculate the ideal sample needed for this study (Faul et al., 2009). In order to detect an effect size *f*^2^ (*V*)=0.06 with 95% power (alpha=0.05), G*Power suggests we would need 208 participants to carry MANOVA analysis. The aim was to recruit a group of participants larger than the ideal sample size in anticipation of potential missing responses or deficient data. Those who responded too quickly were automatically screened out. The sampling procedure resulted in a final sample of 287 students from the faculties of Psychology and Education at the University of Santiago de Compostela (Spain; 85.7% women, age=20.28; *SD*=2.19).

The experimental design was 2 (priming the environmental concern vs. no priming)×2 (type of information: only advantages vs. advantages and disadvantages). All data and materials used in this research are publicly accessible at osf.io/97v45. No studies in this manuscript were preregistered.

### Procedure

Students were asked to participate in a research project that was taking place at the University. To participate, they were required to fill out a questionnaire using the Qualtrics platform on their mobile device or laptop. Participants were randomly assigned to each of the experimental conditions. Participants took an average of 13min to complete the task.

Participants answered a questionnaire that consists of five parts: an introduction, a priming section (with two conditions), an information section (with two conditions), another information section with a series of questions about acceptance of the decentralized plant, and a final section relating to sociodemographic information and debriefing.

The introduction of the questionnaire includes an acknowledgement of their participation and asks participants to be honest in their responses. The introduction states that the Bioethics Committee of the University of Santiago de Compostela has approved the study, guaranteeing anonymity and data protection. Participants could interrupt or abandon their participation at any time if they wished. Before fulfilling the questionnaire, students were required to provide informed consent to participate in the research.

Once the students accepted the commitment, the program randomly assigned the participants to the different experimental conditions. First, participants were told that they would be asked a few questions from another ongoing research project, making additional use of their involvement. This opening allowed the opportunity to present priming information before presenting the decentralized plants information.

#### Priming Conditions

Half of the participants were randomly assigned to one of two conditions: the environmental concern priming group or the control group. In the environmental concern priming group, participants were required to consider environmental problems before they were presented with information about the decentralized plant. To do this, participants answered two questions. First, they were asked to rank a series of environmental challenges by importance: climate change, water scarcity, air pollution, water pollution, deforestation, soil degradation, energy consumption, and waste. Next, participants had to rate the degree of importance of those environmental issues from 1 (none) to 9 (a lot).

In the control condition group, to keep the participants as cognitively active as those in the experimental group, participants were required to order a series of musical styles by affinity. They were then asked to indicate how much they like each of these musical styles, from 1 (none) and 9 (a lot).

#### Information Conditions

In the next section, participants were shown a message that thanked them for their participation in the other investigation and informed them that they were to answer the investigation questions for the second study. In this section, they were required to imagine that their faculty was developing a project to install a plant to treat wastewater in the faculty’s basement. Participants were provided an explanation as to the plant’s functioning. Then, participants received a set of randomized information. Half of the participants read information about the plant that presented its advantages, while the other half read information about the advantages and the possible disadvantages of the plant (see the Annex for complete information).

Next, all participants were required to answer the substantive questions. The purpose of these questions was to determine whether the acceptance of a decentralized wastewater treatment plant differed among the participants after they were randomly exposed to priming and the information presented.

To finalize the questionnaire, participants answered one block of sociodemographic questions. They were then shown a goodbye message, again thanking them for their participation. Participants read that this was a hypothetical situation for research purposes; their faculty would not install a decentralized wastewater treatment plant. Participants could provide their email to obtain a report with the results of the investigation. Furthermore, they were also provided a contact email if they wanted to report, solve, or discuss any issue about the research project.

### Measures

We used several different types of measures to determine the level of acceptance of decentralized plants: attitudes, strength of attitudes, emotions, and behavioral intention.

#### Attitudes Toward Decentralized Plants

Participants were required to answer on a 9-point semantic differential scale (1=nothing and 9=a lot) to what extent they thought that the faculty’s decentralized plant project was: “very bad-very good,” “I do not like it at all-I like it very much,” “very negative-very positive,” “very unnecessary-very necessary,” “very useless-very useful,” “very unacceptable-very acceptable,” “very inappropriate-very appropriate,” or “extremely harmful-extremely beneficial” (*α*=0.91).

#### Strength of Attitudes

Participants assessed their opinions about the installation of the plant in the faculty. On a 9-point semantic differential scale (1=nothing and 9=a lot), they had to answer additional questions about their previous answers including how convinced they were about their opinions, how confident they were in their answers, the relevance of their answers, and how easily they would change their opinion in a discussion (*α*=0.87).

#### Emotions

Participants responded to what extent thinking about the installation of the plant in the faculty makes them feel a number of emotions (1=nothing and 9=a lot): worried, disgusted, angry, fearful, helpless (negative emotions, α=0.78), relieved, proud, optimistic, enthusiastic, and comfortable (positive emotions, *α*=0.84).

#### Behavioral Intention

Participants indicated their degree of agreement (1=no agreement and 9=totally agree) with the following statements: they would support the installation of the plant in the faculty, they would campaign in favor of the installation of the plant in the faculty, they would recommend that these plants be installed in other buildings of the University and the city, and they would install a plant in their building or house (*α*=0.88).

### Priming Control

In order to draw valid conclusions, participants should not identify the connection between the priming task and the subsequent task (Bargh, 2006). In this study, participants had to answer an open question asking them what they believed the objective of the research is.

## Results

In the open response question, none of the participants identified the relationship between both tasks. Participants referred to questions about “assessing/checking the degree of acceptance of the technology presented,” “how people perceive a new technology after presenting information about it,” and “assessing opinions that can be controversial anonymously.” No one referred to the effect that the questionnaire’s first task had on the second part of the questionnaire, demonstrating that they were not aware of the priming task.

After verifying that the participants were not aware of the experiment’s manipulation, we analyzed the effect that environmental concern priming had on the acceptance of decentralized plants. Specifically, we considered how the inclusion or exclusion of information about plant disadvantages influenced the participants’ perceptions. Table 1 shows the MANOVA results for each of the variables under study in each of the conditions.

**Table 1:** MANOVA results.

Condition	Variables	Condition level		*M*	*SD*	*F*	Sig.	*η* ^2^
Priming	Attitudes	Control		7.19	1.13	8.10	0.005^**^	0.028
		Environmental		7.48	1.07			
	Attitudes strength	Control		6.06	1.54	9.97	0.002^**^	0.034
		Environmental		6.64	1.55			
	Behavioral intention	Control		7.09	1.44	6.32	0.013^**^	0.022
		Environmental		7.50	1.32			
	Positive emotions	Control		6.06	1.34	8.14	0.005^**^	0.028
		Environmental		6.53	1.43			
	Negative emotions	Control		3.02	1.41	0.73	0.394	0.003
		Environmental		2.87	1.47			
Information	Attitudes	Advantages		7.54	1.10	7.27	0.007^**^	0.025
		Advantages+disadvantages		7.19	1.10			
	Attitudes strength	Advantages		6.41	1.55	0.29	0.589	0.001
		Advantages+disadvantages		6.30	1.60			
	Behavioral intention	Advantages		7.45	1.40	3.53	0.061	0.013
		Advantages+disadvantages		7.14	1.38			
	Positive emotions	Advantages		6.25	1.53	0.44	0.501	0.002
		Advantages+disadvantages		6.36	1.26			
	Negative emotions	Advantages		2.75	1.37	5.33	0.022^*^	0.019
		Advantages+disadvantages		3.15	1.49			
Priming x information	Attitudes	Control	Advantages	7.46	1.08	2.91	0.089	0.010
			Advantages+disadvantages	6.90	1.12			
		Environmental	Advantages	7.61	1.11			
			Advantages+disadvantages	7.48	1.02			
	Strength of attitudes	Control	Advantages	6.08	1.61	0.090	0.764	0.001
			Advantages+disadvantages	6.04	1.48			
		Environmental	Advantages	6.72	1.43			
			Advantages+disadvantages	6.56	1.68			
	Behavioral intention	Control	Advantages	7.27	1.44	0.105	0.746	0.001
			Advantages+disadvantages	6.91	1.44			
		Environmental	Advantages	7.63	1.34			
			Advantages+disadvantages	7.37	1.28			
	Positive emotions	Control	Advantages	6.05	1.53	0.294	0.588	0.001
			Advantages+disadvantages	6.07	1.12			
		Environmental	Advantages	6.43	1.51			
			Advantages+disadvantages	6.63	1.33			
	Negative emotions	Control	Advantages	2.92	1.42	1.17	0.280	0.004
			Advantages+disadvantages	3.13	1.41			
		Environmental	Advantages	2.59	1.30			
			Advantages+disadvantages	3.17	1.57			

** *p<0.01;*

* *p<0.05.*

As one can see in Table 1, the effect of priming is significant. Having participants think about environmental issues before being presented the information about decentralized plants affected their level of acceptance. Thus, those participants who received the environmental concern priming obtained significantly higher scores than the control group in the measures: attitudes (*F*=8.10, *p*=0.005, *η*^2^=0.028), strength of attitudes (*F*=9.97, *p*=0.002, *η*^2^=0.034), behavioral intention (*F*=6.32, *p*=0.013, *η*^2^=0.022), and positive emotions (*F*=8.14, *p*=0.005, *η*^2^=0.028). There were no significant differences between the control group and the experimental group regarding negative emotions (*F*=0.73, *p*=0.394, *η*^2^=0.003).

Regarding the informative content of the message, presenting the advantages and disadvantages of the plant produced attitudes that were significantly more negative than those who only received information about the advantages (*F*=7.27, *p*=0.007, *η*^2^=0.025). Those who read information about disadvantages experienced slightly stronger negative emotions than those who read only advantages (*F*=5.33, *p*=0.022, *η*^2^=0.19). However, reporting advantages and disadvantages did not create significant differences in the strength of attitudes (*F*=0.29, *p*=0.589, *η*^2^=0.001), behavioral intention (*F*=3.53, *p*=0.061, *η*^2^=0.013), or positive emotions measures (*F*=0.44, *p*=0.501, *η*^2^=0.002).

The interaction of the two conditions (i.e., the priming task and type of information) was not significant for any of the variables under study.

## Discussion

Decentralized wastewater treatment plants allow recovery and reuse of water and nutrients from wastewater, promoting the circular economy (Lens et al., 2005; Roefs et al., 2017). Although this technology may be a possible solution to the global water crisis, the truth is that implementation of the technology depends on having social acceptance (Mankad, 2012; Gómez-Román et al., 2020).

Although numerous studies have shown that providing information is a facilitating factor for acceptance (Mankad and Tapsuwan, 2011; Rolland et al., 2020), the way such information is presented is not a trivial question. It can have decisive consequences for the development of public opinion (Valentin and Bogus, 2015). How that message is presented is key to gaining broad consensus (Wiest et al., 2015). Consequently, the communication processes in the formation of interpretive frameworks on this technology are critical, especially when public opinion on this technology is not yet clear.

In this study, our goal was to determine whether asking people to think about environmental problems (through environmental concern priming) before presenting information about decentralized wastewater treatment plants influences their acceptance of the technology. Moreover, we wanted to test whether including bilateral arguments about the technology’s disadvantages influenced the degree of acceptance.

The results partially confirm the hypotheses of this study. As expected, those who think about environmental problems before receiving the information about plants had a more positive perception of the technology. However, the effect of presenting unilateral or bilateral arguments is less clear. Mentioning only the plants’ advantages led to more positive attitudes and fewer negative emotions relating to these technologies, but there were no significant differences in the rest of the acceptance indicators. Although the trend in the acceptance indicators, strength of attitudes, and behavioral intention were similar, those who received arguments only about the advantages had a more positive perception of the technology. That being said, the difference was not significant compared to those who received information about both the advantages and disadvantages.

Even though the priming and information interaction was not significant, acceptance was more favorable even where the disadvantages were presented so long as participants were primed through questions about environmental concern. As expected, environmental concern priming reduced differences in acceptance between those who received information about advantages only and those who received information about both the advantages and disadvantages. Therefore, activating environmental concern improved participants’ perception of information about decentralized wastewater treatment plants, even when the technology’s disadvantages were explicitly presented.

Considering that this is an exploratory study, these results need to be considered cautiously, and they are only an initial approximation. Firstly, because of the sample, the study relied on university students as participants to test this exploratory hypothesis. Now there is a need to replicate these results in a more general population sample, also in different contexts. Secondly, because the effect sizes were relatively small, so future studies should replicate these findings to make stronger statements about the trend of this exploratory study. Although the effect sizes found were modest, this research provides encouraging evidence for its value as an explanatory mechanism to launch communication campaigns and catalyze other research studies with larger samples. The purpose of this research implies that it is necessary to examine public opinion about an issue that is not yet up for debate on the public agenda. It is necessary to consider carefully how we present the information even in the control condition. The first impression on a new topic establishes the framework from which one will process the rest of the information on that issue (Wilson et al., 1989, 2000), so it is necessary to be cautious when launching a broader population study. Moreover, when doing so, researchers must use “debriefing” strategies to avoid unleashing a possible social debate on the topic that is not yet on the public agenda.

The results of this study indicate that, although the unilateral condition pertaining to advantages with environmental priming shows the most promising results, there were no significant differences between the unilateral and bilateral information scores in the condition of environmental concern priming. In both cases, results were very positive. One can thus conclude that environmental concern priming is a necessary element in improving social acceptance of decentralized wastewater treatment plants. However, it is not as straightforward if the type of arguments presented (i.e., only advantages or also the disadvantages of the technology) play a role. Perhaps the key question is not the type of arguments that are presented, but who provides the arguments (depending on the trust or credibility given to the source). Alternatively, audience characteristics may be critical. Therefore, these questions should be explored (and even combined) to determine which elements are critical when encouraging acceptance of these technologies.

This study is a first step to demonstrate experimentally that acceptance of decentralized wastewater treatment plants depends not only on reporting the qualities of this technology but also on providing the information within the context of global environmental problems.

## Data Availability Statement

All data and materials used in this research are publicly accessible at osf.io/97v45.

## Ethics Statement

The studies involving human participants were reviewed and approved by Bioethics Committee of the University of Santiago de Compostela. The patients/participants provided their written informed consent to participate in this study.

## Author Contributions

J-MS: funding. CG-R, J-MS, and MA: conceptualization and methodology. CG-R and J-MS: writing original draft, review, and editing. MA and BM: writing – review and editing. All authors contributed to the article and approved the submitted version.

## Funding

This project had received funding from the European Union’s Horizon 2020 Research and Innovation Program under grant agreement no. 730285. The authors belong to the Galician Competitive Research Group GRC/GPC2016-017-GI-1456, COSOYPA, and CRETUS Strategic Partnership (AGRUP2015/02). All these programs are co-funded by FEDER (UE).

## Conflict of Interest

The authors declare that the research was conducted in the absence of any commercial or financial relationships that could be construed as a potential conflict of interest.

Publisher’s Note: All claims expressed in this article are solely those of the authors and do not necessarily represent those of their affiliated organizations, or those of the publisher, the editors and the reviewers. Any product that may be evaluated in this article, or claim that may be made by its manufacturer, is not guaranteed or endorsed by the publisher.

## Annex

### Information about decentralized wastewater treatment plants (advantages and disadvantages)

Imagine that the Faculty has a project to install a plant to treat the wastewater in the Faculty’s basement.

Until now, the wastewater from the Faculty and the rest of the buildings and houses in Santiago are channeled through the sewerage networks to the centralized treatment plant in Silvouta, just over 6km from the city center.

The Faculty is proposing treating *in situ* the wastewater in the building. That is, the different types of water: gray (from the sinks) and black (from the toilets) that are generated in the Faculty would be collected separately and once treated and purified in the basement floor, used for different uses, such as filling cisterns or watering green areas.

That would save a large amount of drinking water. Each time the cistern is flushed, 8–10 liters of drinking water are used. Considering the number of people who work/study at the Faculty, that would mean saving about 19,200 liters of drinking water every day.

Another advantage is that this plant would recover the phosphorus in the wastewater and use it as fertilizer. Phosphorous is a rare mineral, which is why it has become a strategic priority for food production.

Nevertheless, that plant also has some drawbacks. One of them is that every now and then, and due to failure, it can produce unpleasant odors.

Its installation would also entail a significant economic cost that the Faculty would have to be borne to build a new pipes system that would separate the gray water from the black, to build the plant, and to maintain it.
